# Supplementary material for: EPHX1 mutations cause a lipoatrophic diabetes syndrome due to impaired epoxide hydrolysis and increased cellular senescence
Source: eLife. 2021 Aug 3;10:e68445. doi: 10.7554/eLife.68445 (PMC8331186; doi:10.7554/eLife.68445)
Supplement: Source data 1. [file elife-68445-data1.zip › unedited blot marked/Figure 4-fig suppl 4 unedited western blot source data.pptx]

## Slide 1
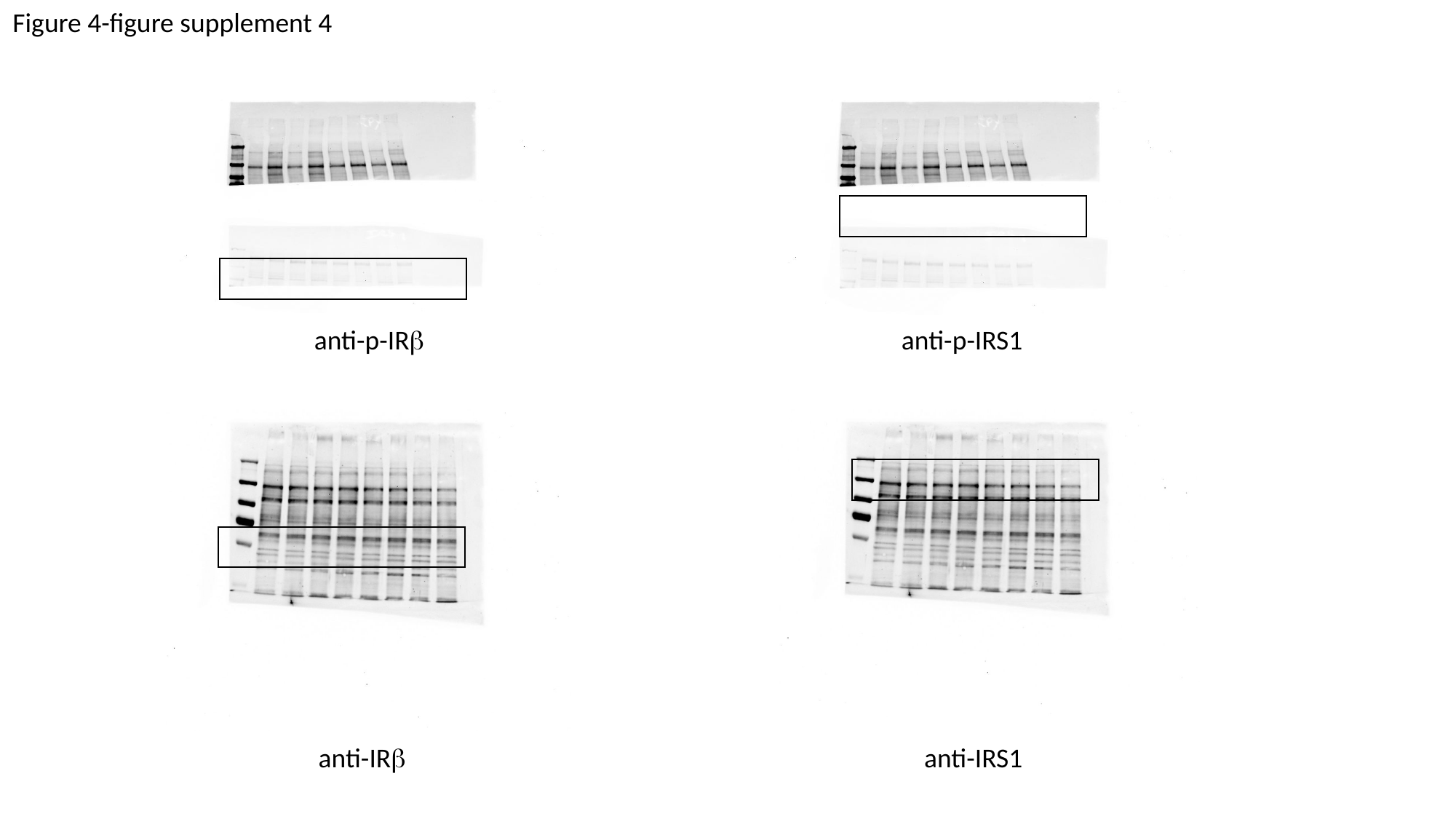

Figure 4-figure supplement 4
anti-p-IRb
anti-p-IRS1
anti-IRb
anti-IRS1

## Slide 2
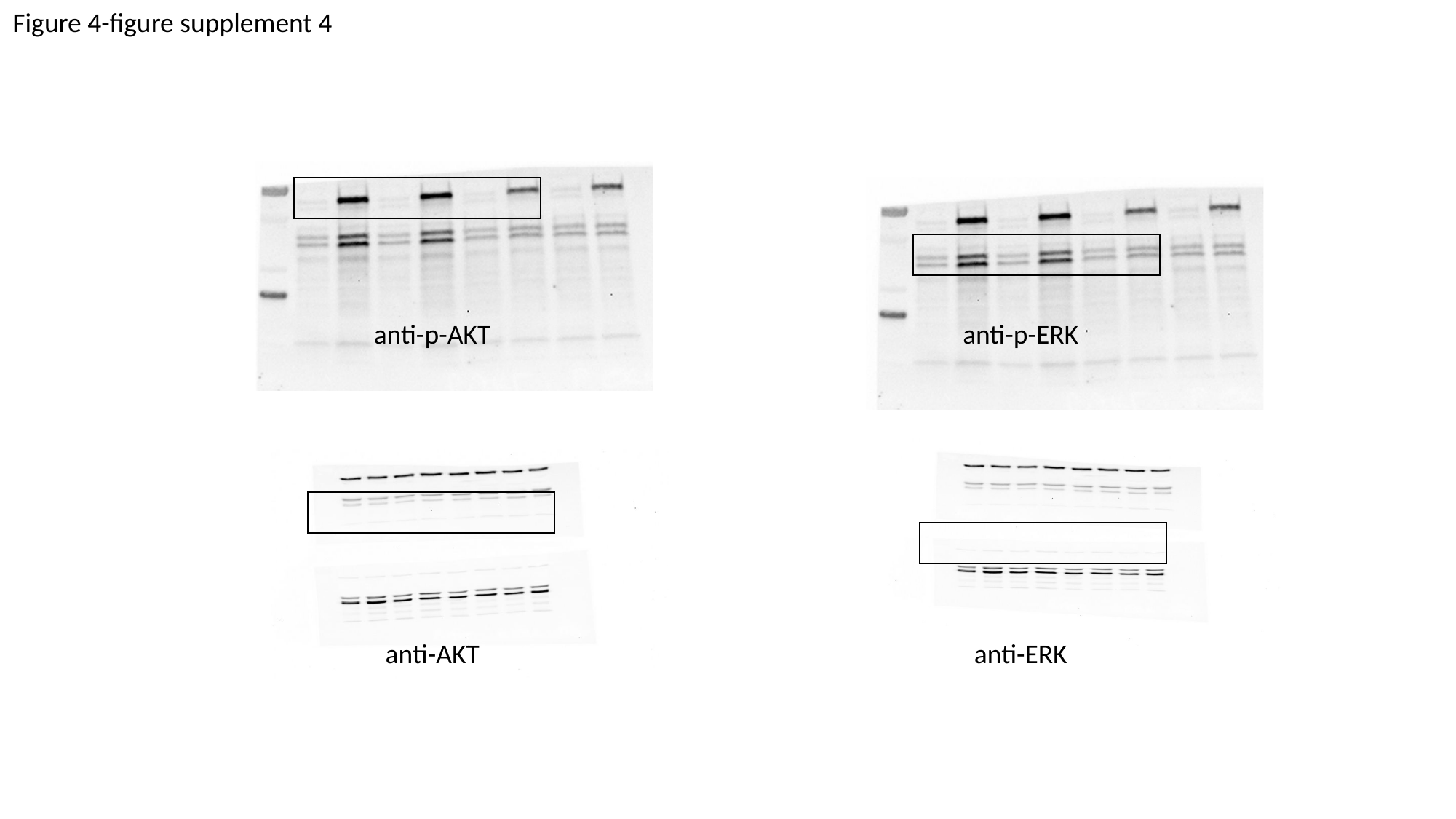

Figure 4-figure supplement 4
anti-p-AKT
anti-p-ERK
anti-AKT
anti-ERK
