# Supplementary figures and images for: EPHX1 mutations cause a lipoatrophic diabetes syndrome due to impaired epoxide hydrolysis and increased cellular senescence
Source: eLife. 2021 Aug 3;10:e68445. doi: 10.7554/eLife.68445 (PMC8331186; doi:10.7554/eLife.68445)

## Slide 1
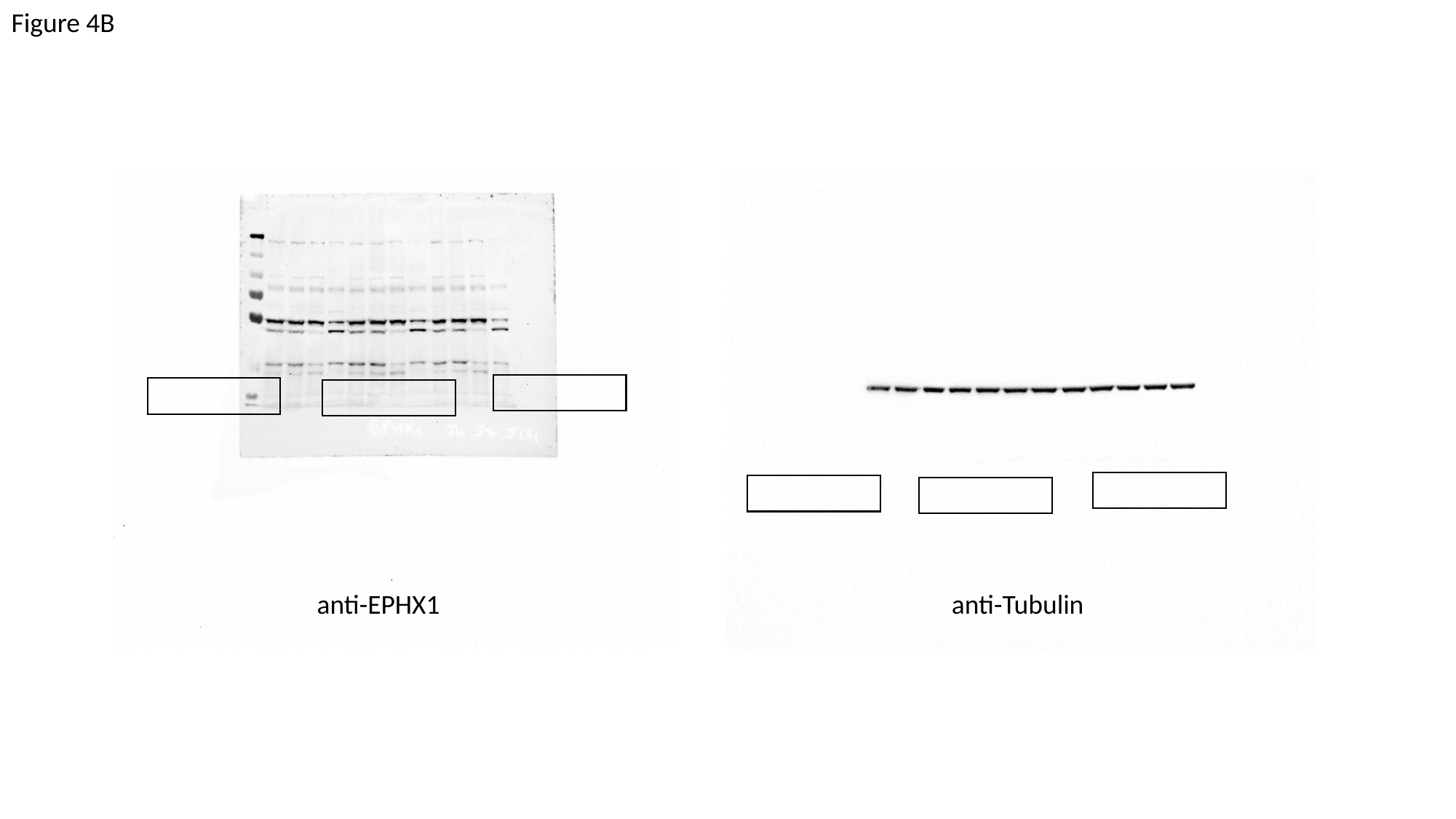

Figure 4B
anti-EPHX1
anti-Tubulin

Supplement: Source data 1. [file elife-68445-data1.zip › unedited blot marked/Figure 4B unedited western blot source data.pptx]

## Slide 1
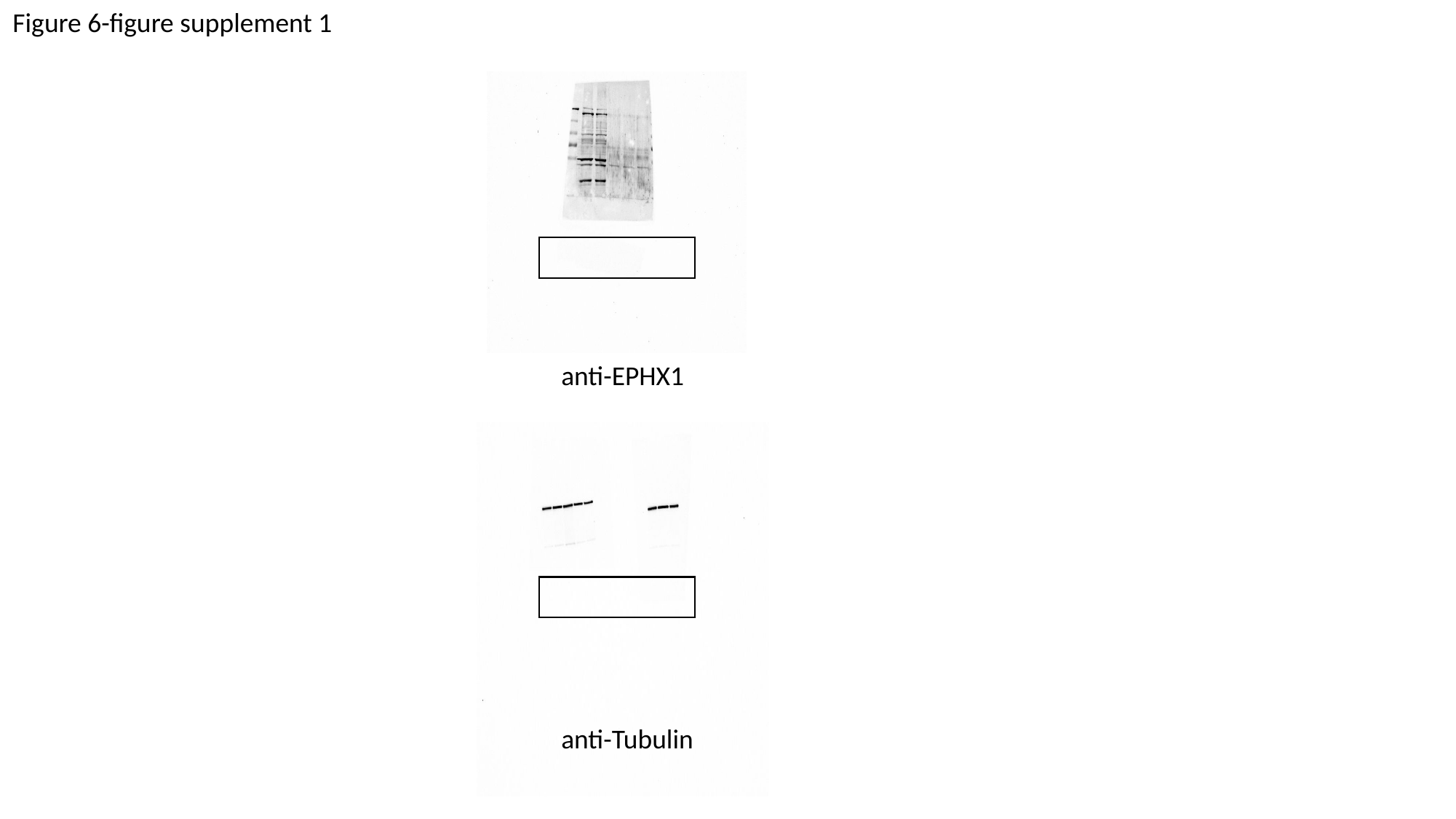

Figure 6-figure supplement 1
anti-EPHX1
anti-Tubulin

Supplement: Source data 1. [file elife-68445-data1.zip › unedited blot marked/Figure 6-fig suppl 1 unedited western blot source data.pptx]

## Slide 1
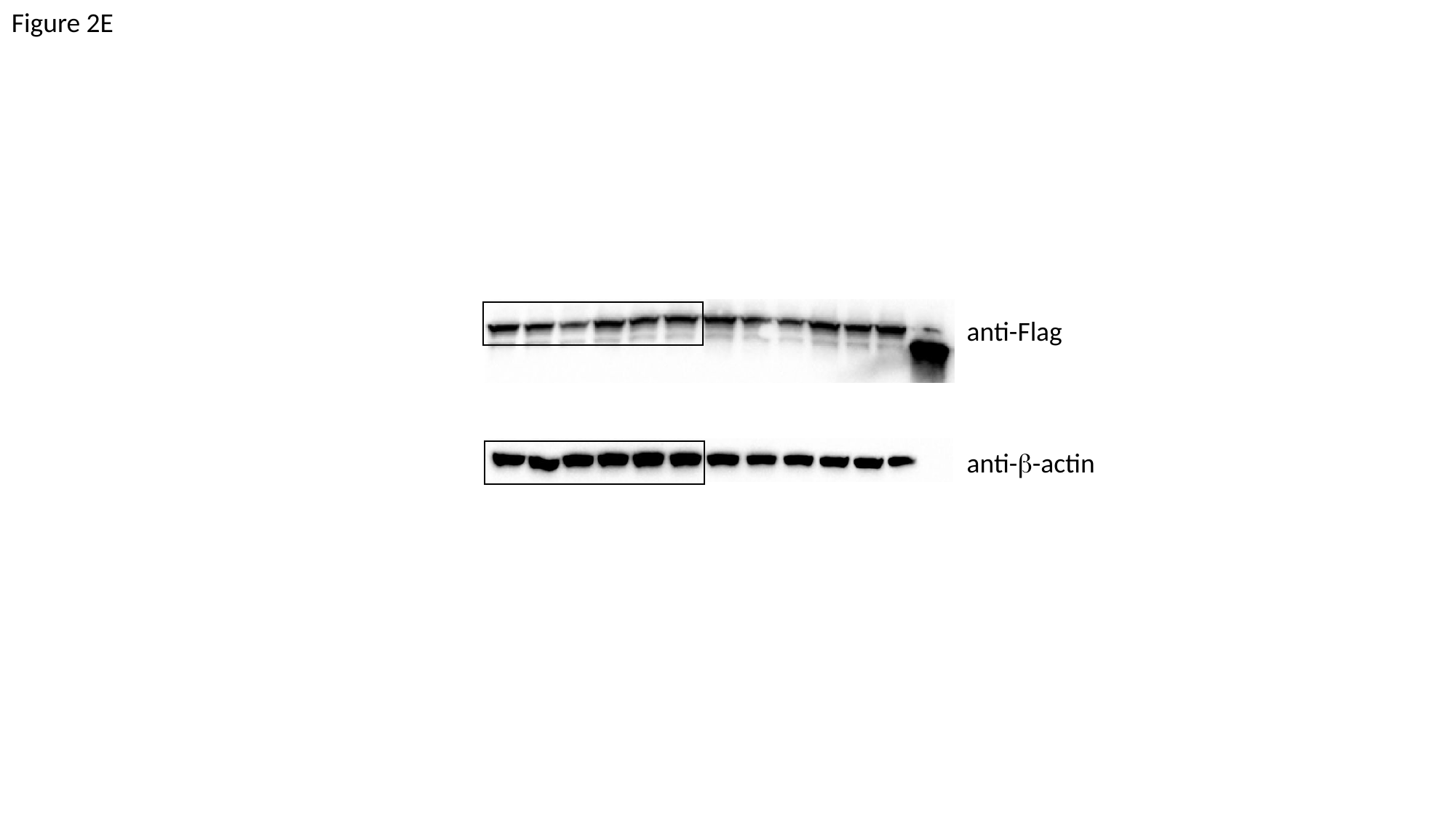

Figure 2E
anti-Flag
anti-b-actin

Supplement: Source data 1. [file elife-68445-data1.zip › unedited blot marked/Figure 2E unedited western blot source data.pptx]

## Slide 1
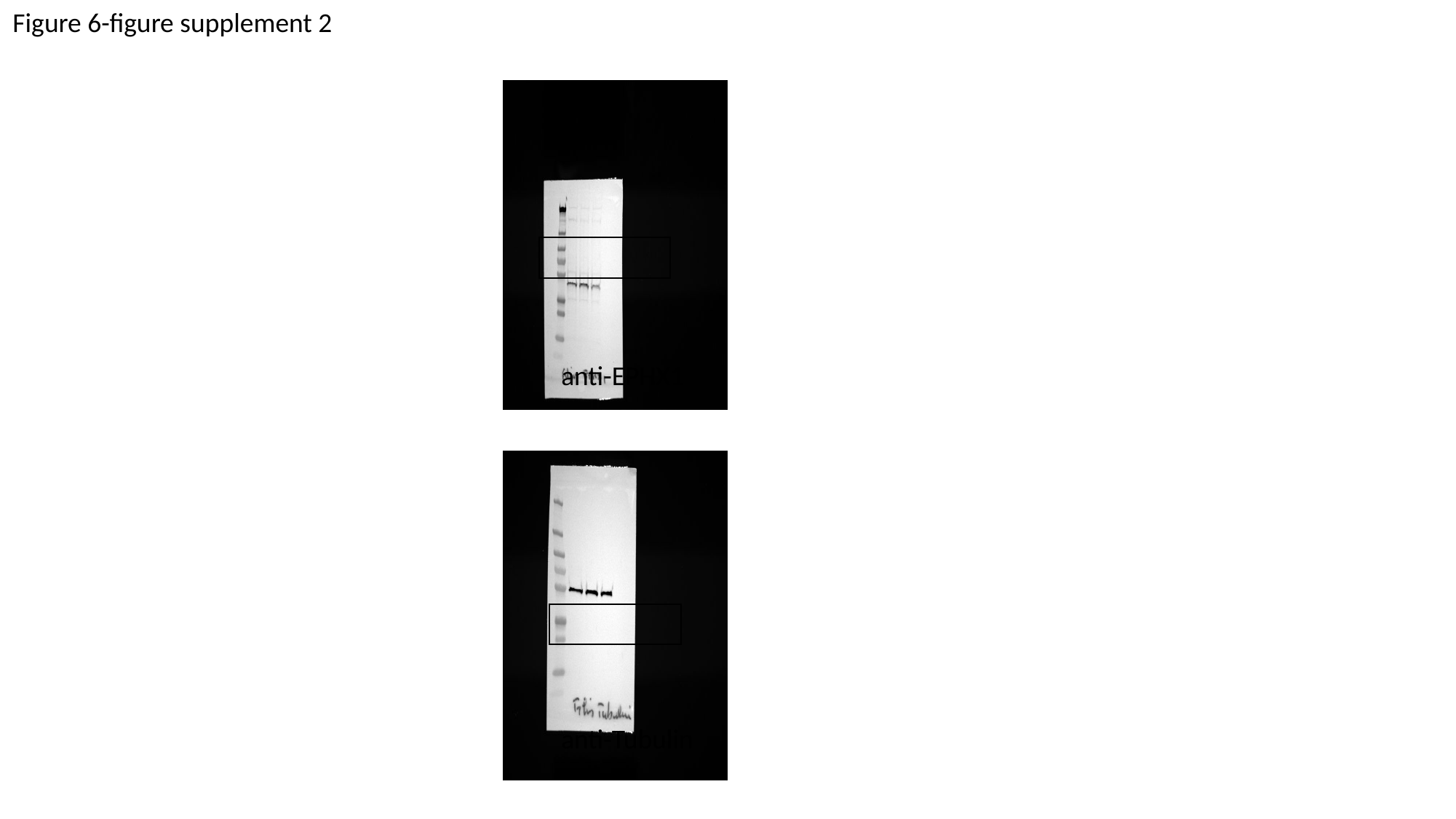

Figure 6-figure supplement 2
anti-EPHX1
anti-Tubulin

Supplement: Source data 1. [file elife-68445-data1.zip › unedited blot marked/Figure 6-fig suppl 2 unedited western blot source data.pptx]

## Slide 1
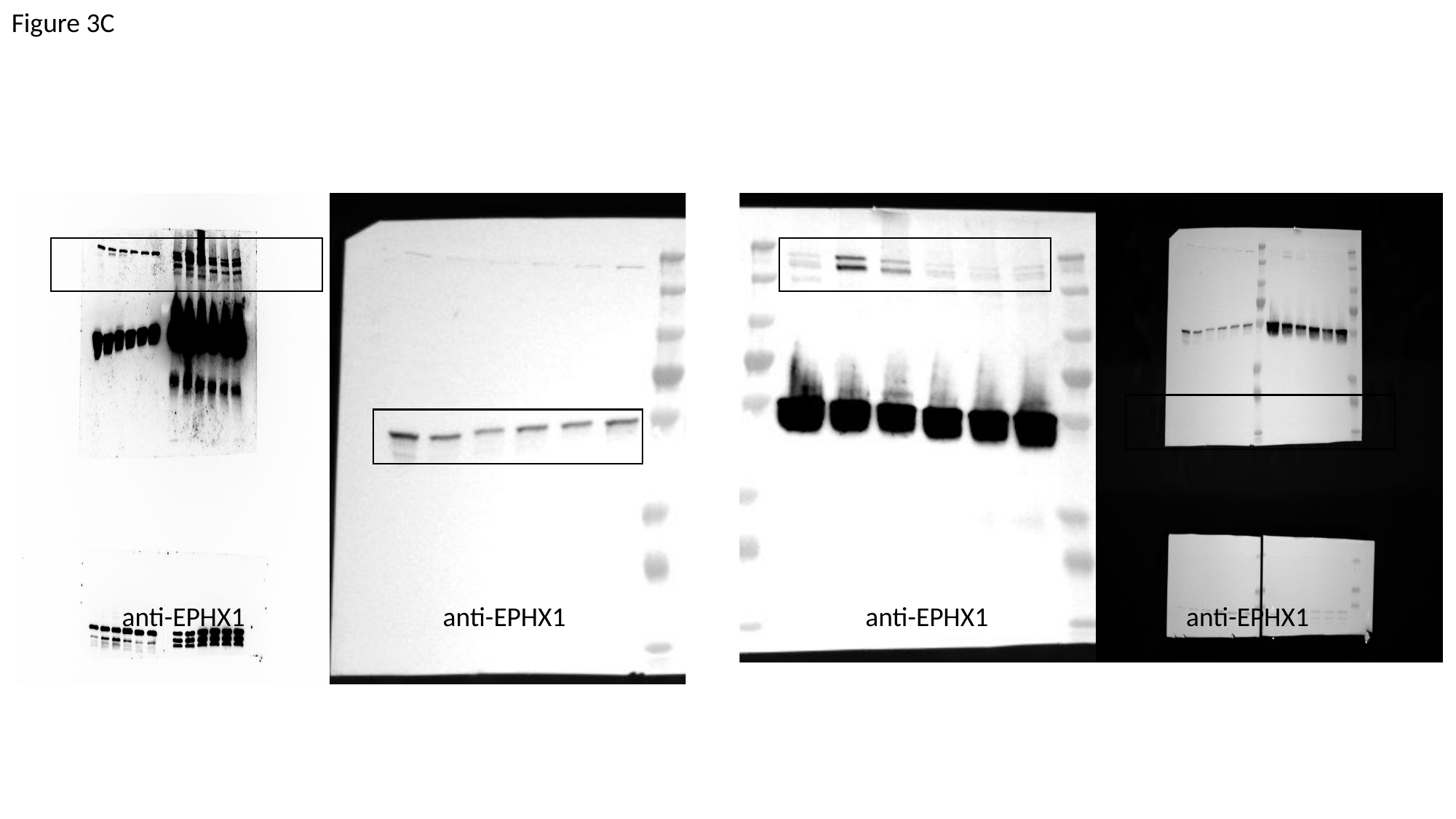

Figure 3C
anti-EPHX1
anti-EPHX1
anti-EPHX1
anti-EPHX1

Supplement: Source data 1. [file elife-68445-data1.zip › unedited blot marked/Figure 3C unedited western blot source data.pptx]

## Slide 1
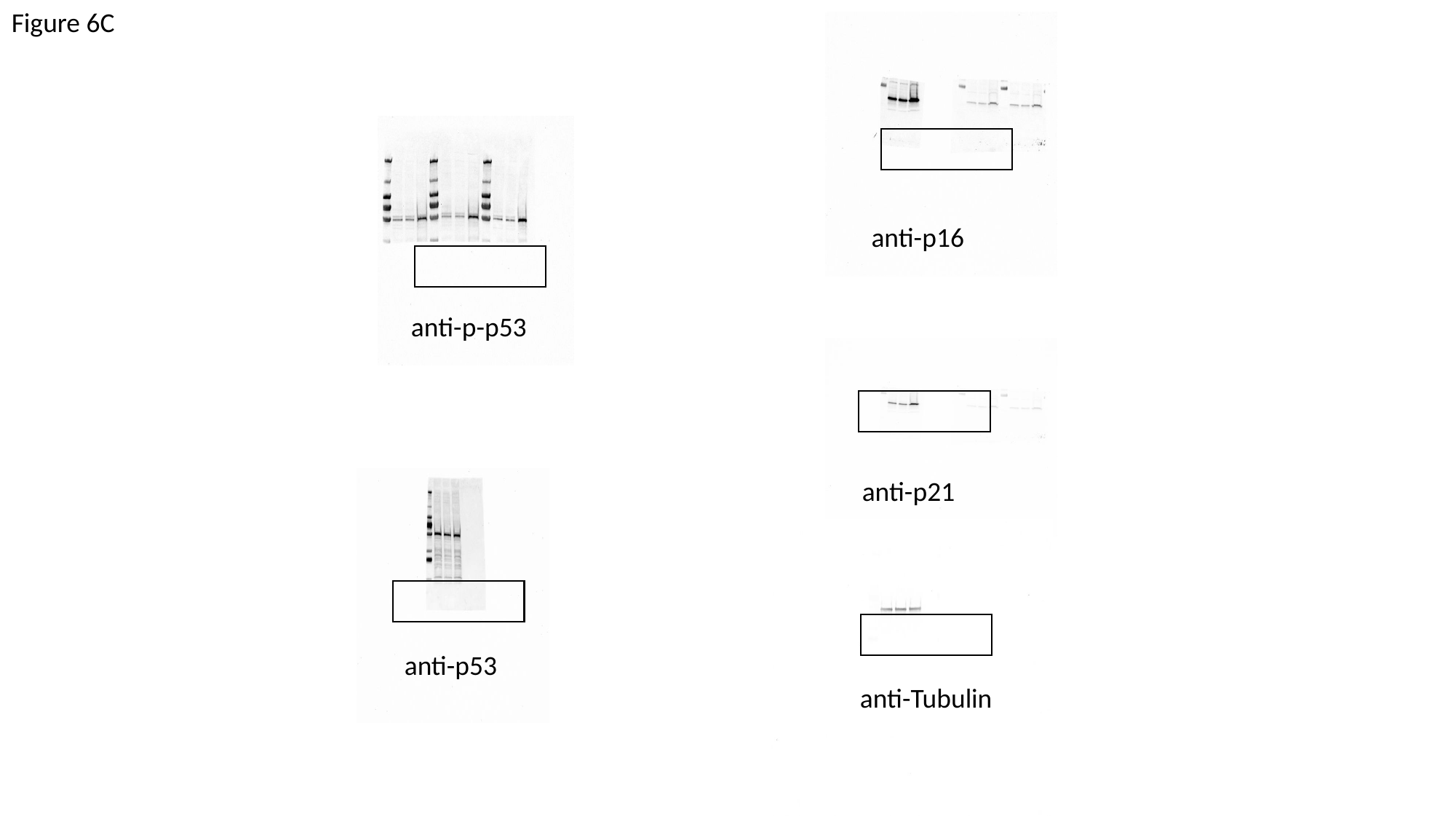

Figure 6C
anti-p16
anti-p-p53
anti-p21
anti-p53
anti-Tubulin

Supplement: Source data 1. [file elife-68445-data1.zip › unedited blot marked/Figure 6C unedited western blot source data.pptx]

## Slide 1
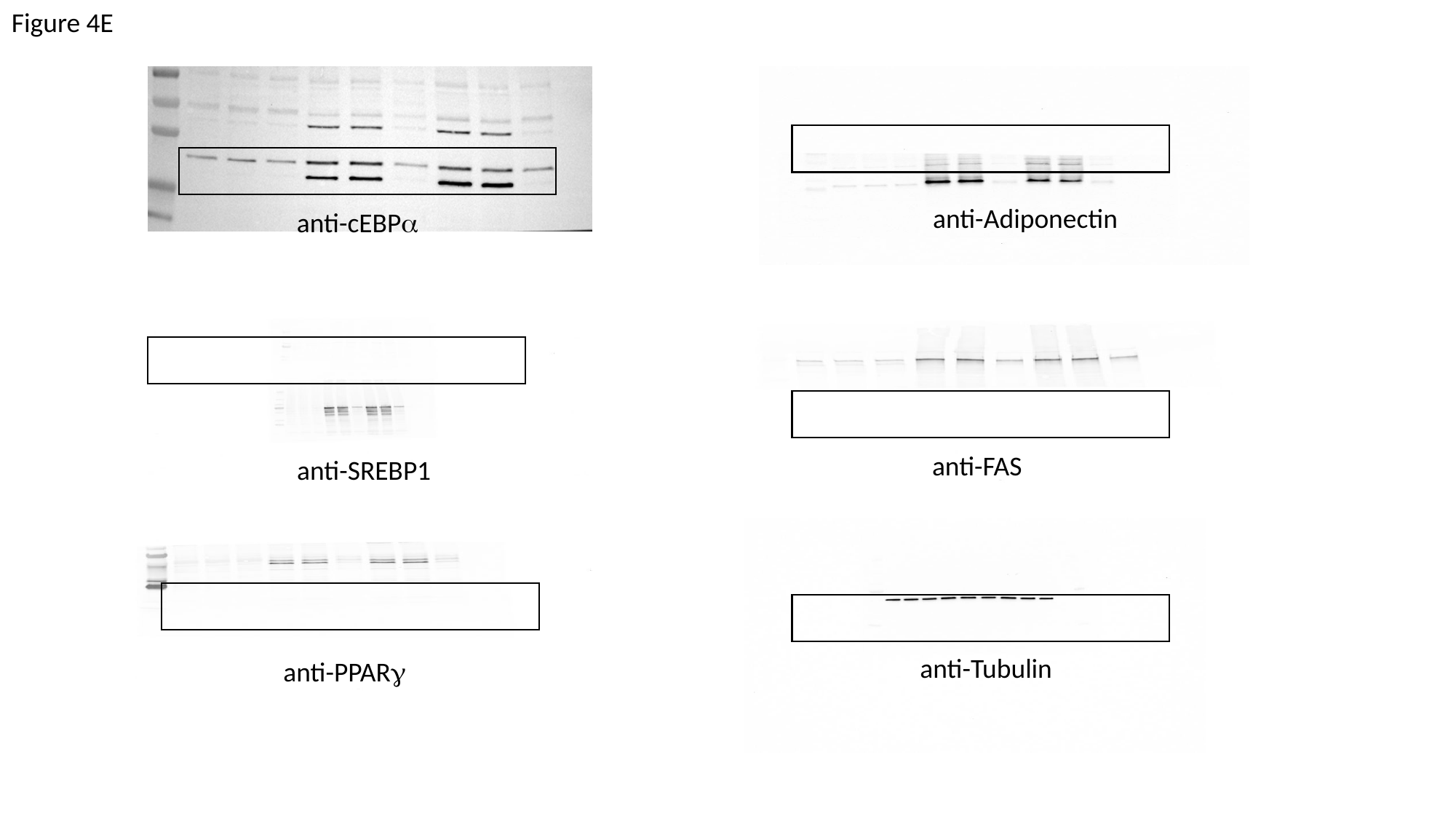

Figure 4E
anti-Adiponectin
anti-cEBPa
anti-FAS
anti-SREBP1
anti-Tubulin
anti-PPARg

Supplement: Source data 1. [file elife-68445-data1.zip › unedited blot marked/Figure 4E unedited western blot source data.pptx]

## Slide 1
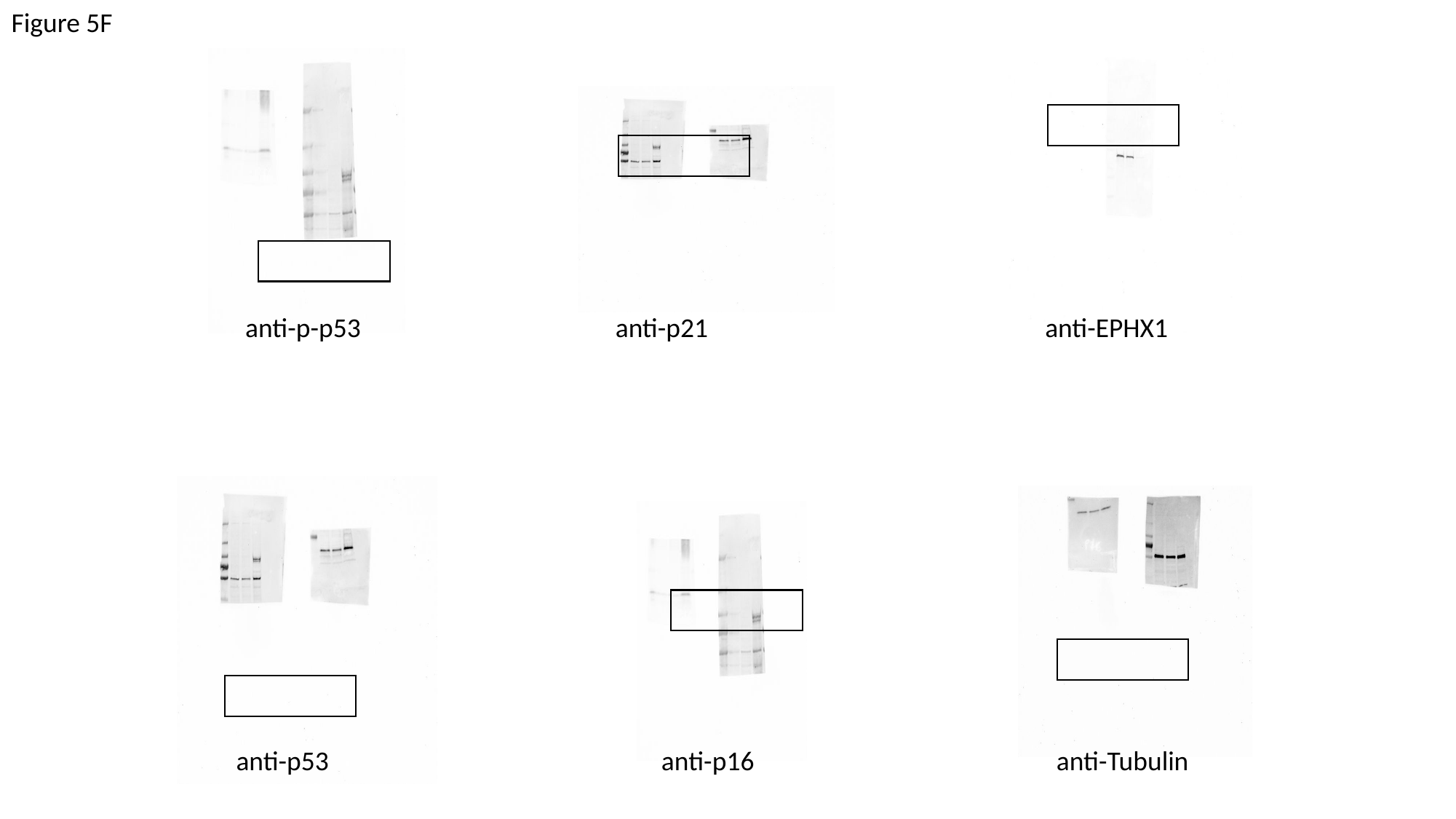

Figure 5F
anti-p-p53
anti-p21
anti-EPHX1
anti-p53
anti-p16
anti-Tubulin

Supplement: Source data 1. [file elife-68445-data1.zip › unedited blot marked/Figure 5F unedited western blot source data.pptx]

## Slide 1
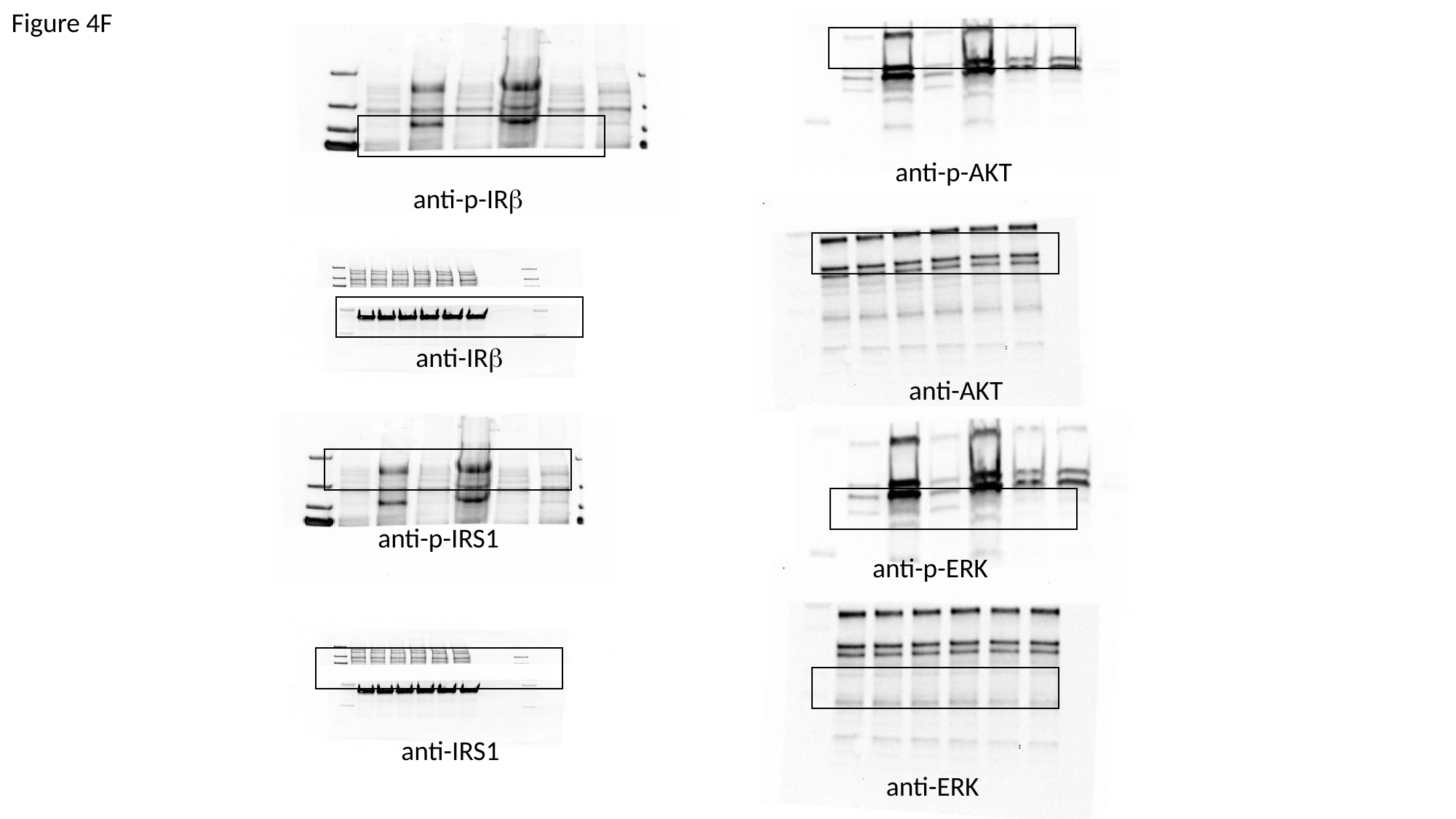

Figure 4F
anti-p-AKT
anti-p-IRb
anti-IRb
anti-AKT
anti-p-IRS1
anti-p-ERK
anti-IRS1
anti-ERK

Supplement: Source data 1. [file elife-68445-data1.zip › unedited blot marked/Figure 4F unedited western blot source data.pptx]

## Slide 1
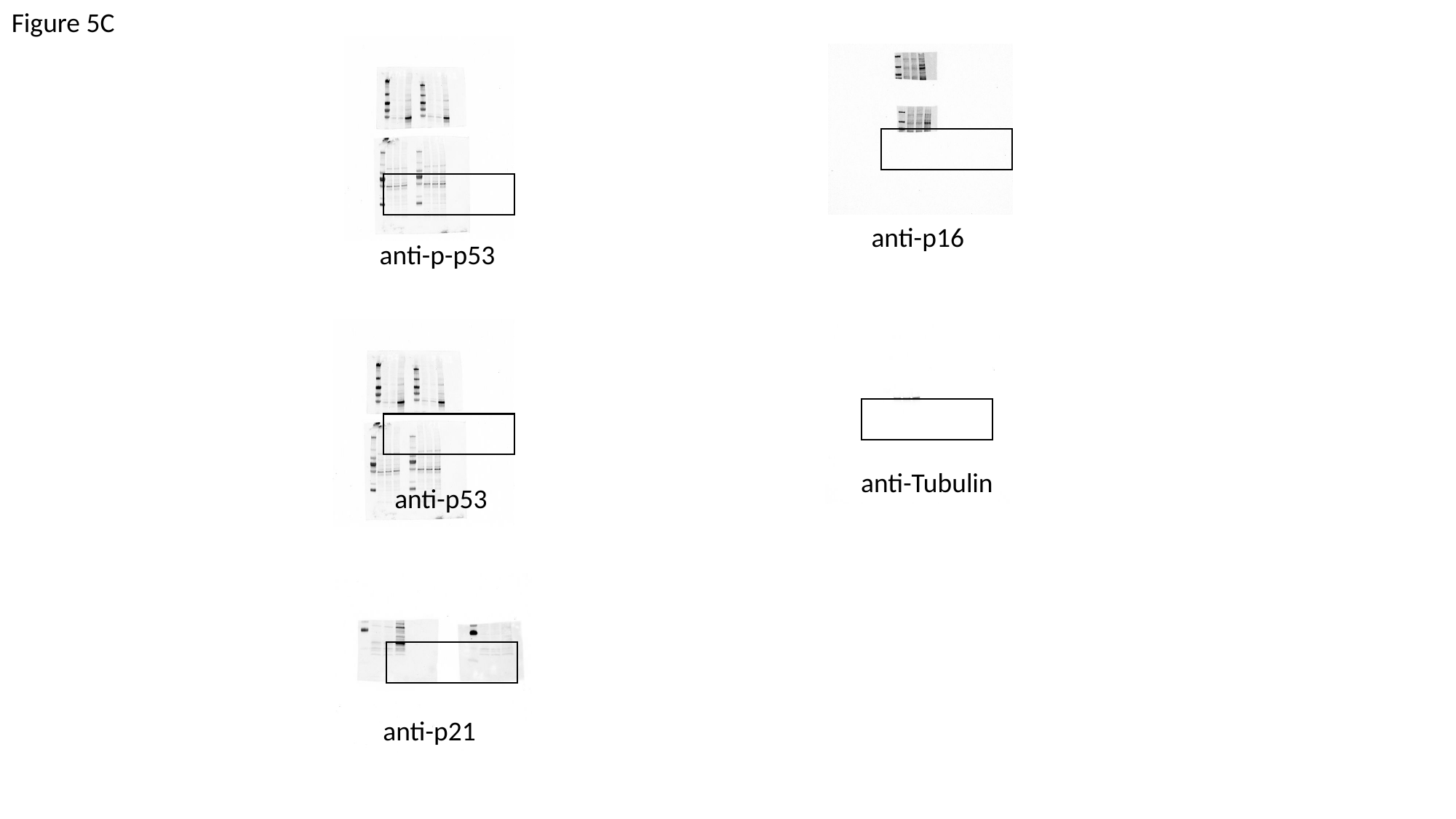

Figure 5C
anti-p16
anti-p-p53
anti-Tubulin
anti-p53
anti-p21

Supplement: Source data 1. [file elife-68445-data1.zip › unedited blot marked/Figure 5C unedited western blot source data.pptx]

## Slide 1
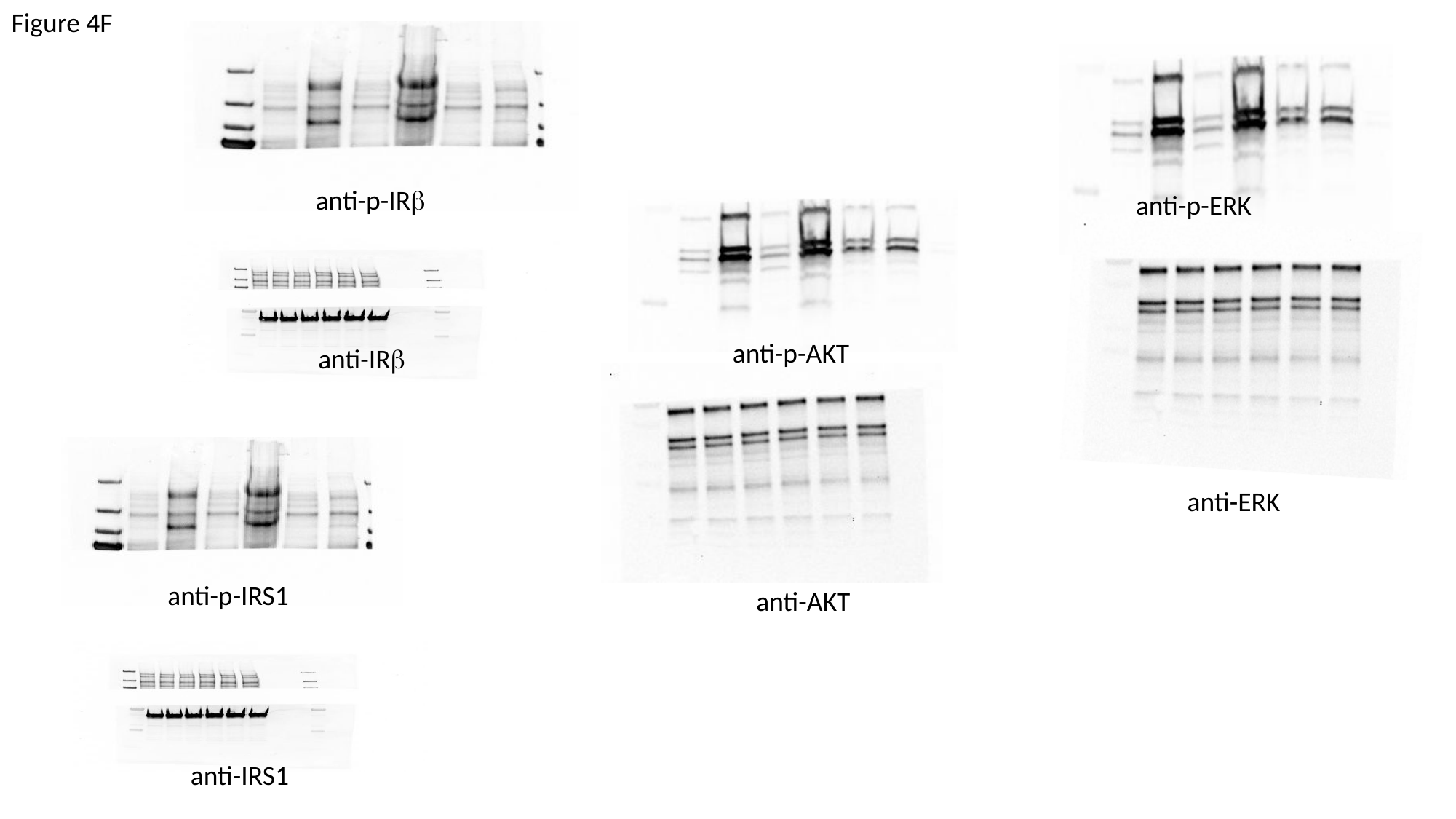

Figure 4F
anti-p-IRb
anti-p-ERK
anti-p-AKT
anti-IRb
anti-ERK
anti-p-IRS1
anti-AKT
anti-IRS1

Supplement: Source data 2. [file elife-68445-data2.zip › unedited blot unmarked/Figure 4F unedited western blot source data unmarked.pptx]

## Slide 1
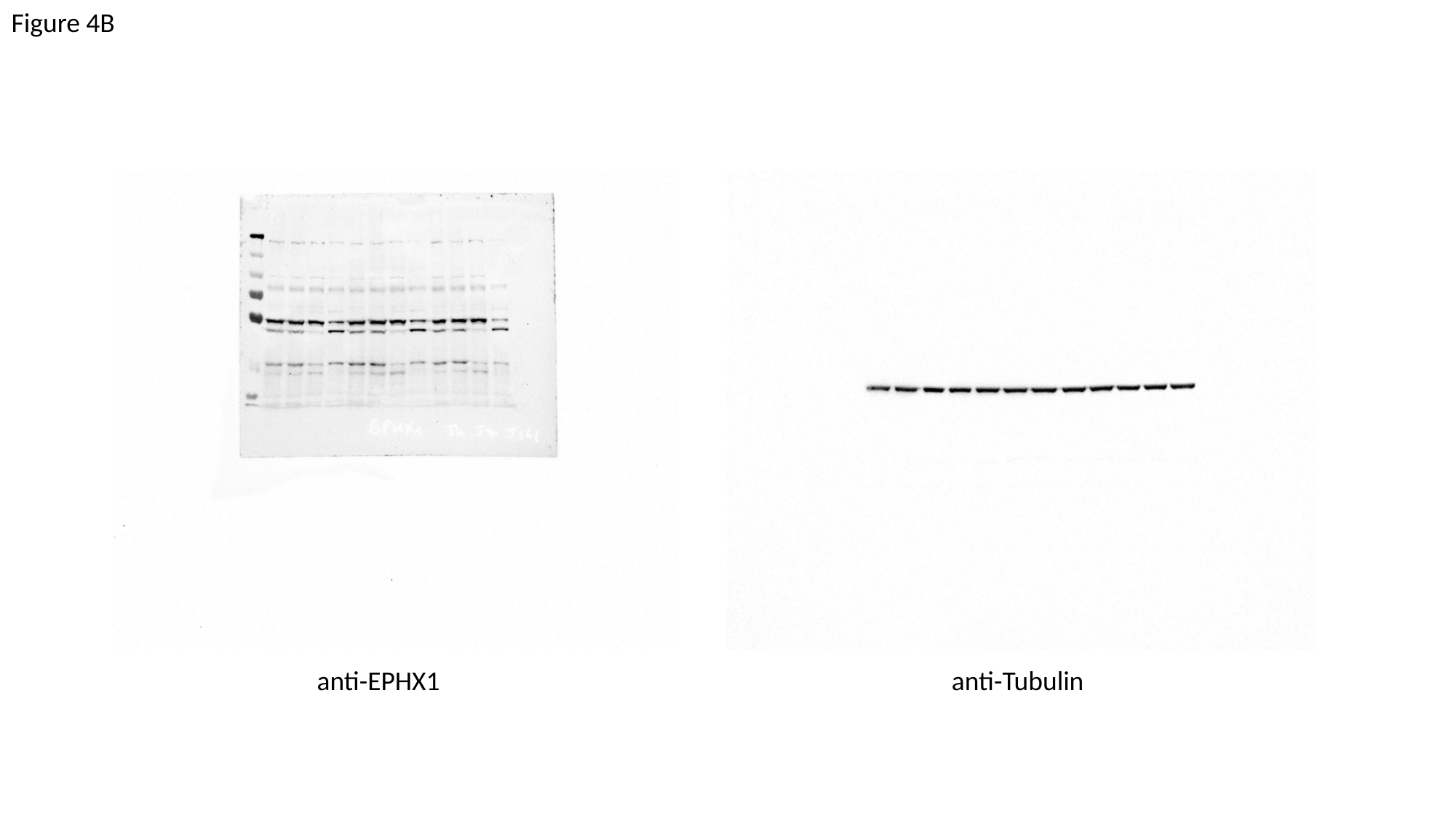

Figure 4B
anti-EPHX1
anti-Tubulin

Supplement: Source data 2. [file elife-68445-data2.zip › unedited blot unmarked/Figure 4B unedited western blot source data unmarked.pptx]

## Slide 1
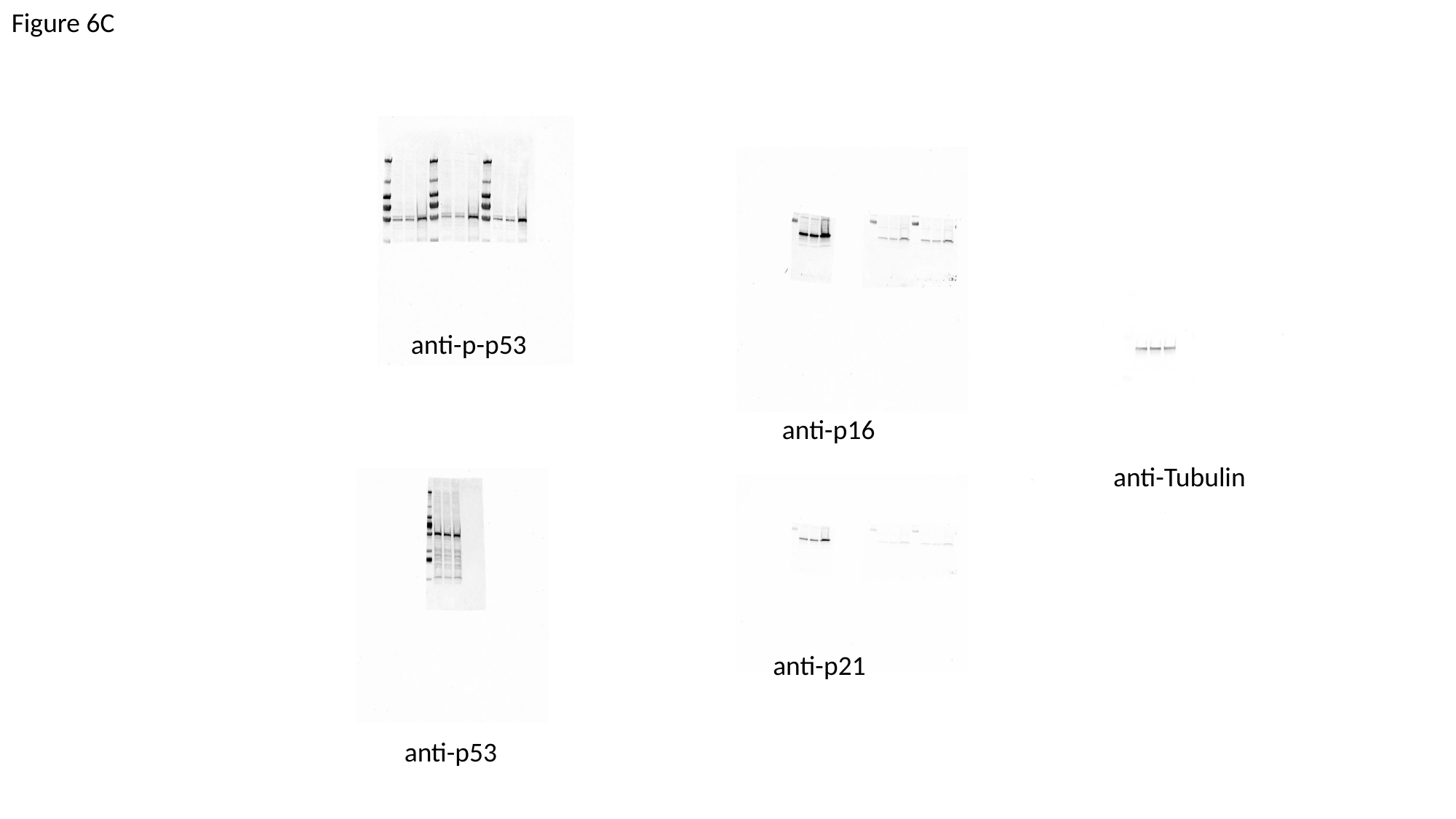

Figure 6C
anti-p-p53
anti-p16
anti-Tubulin
anti-p21
anti-p53

Supplement: Source data 2. [file elife-68445-data2.zip › unedited blot unmarked/Figure 6C unedited western blot source data unmarked.pptx]

## Slide 1
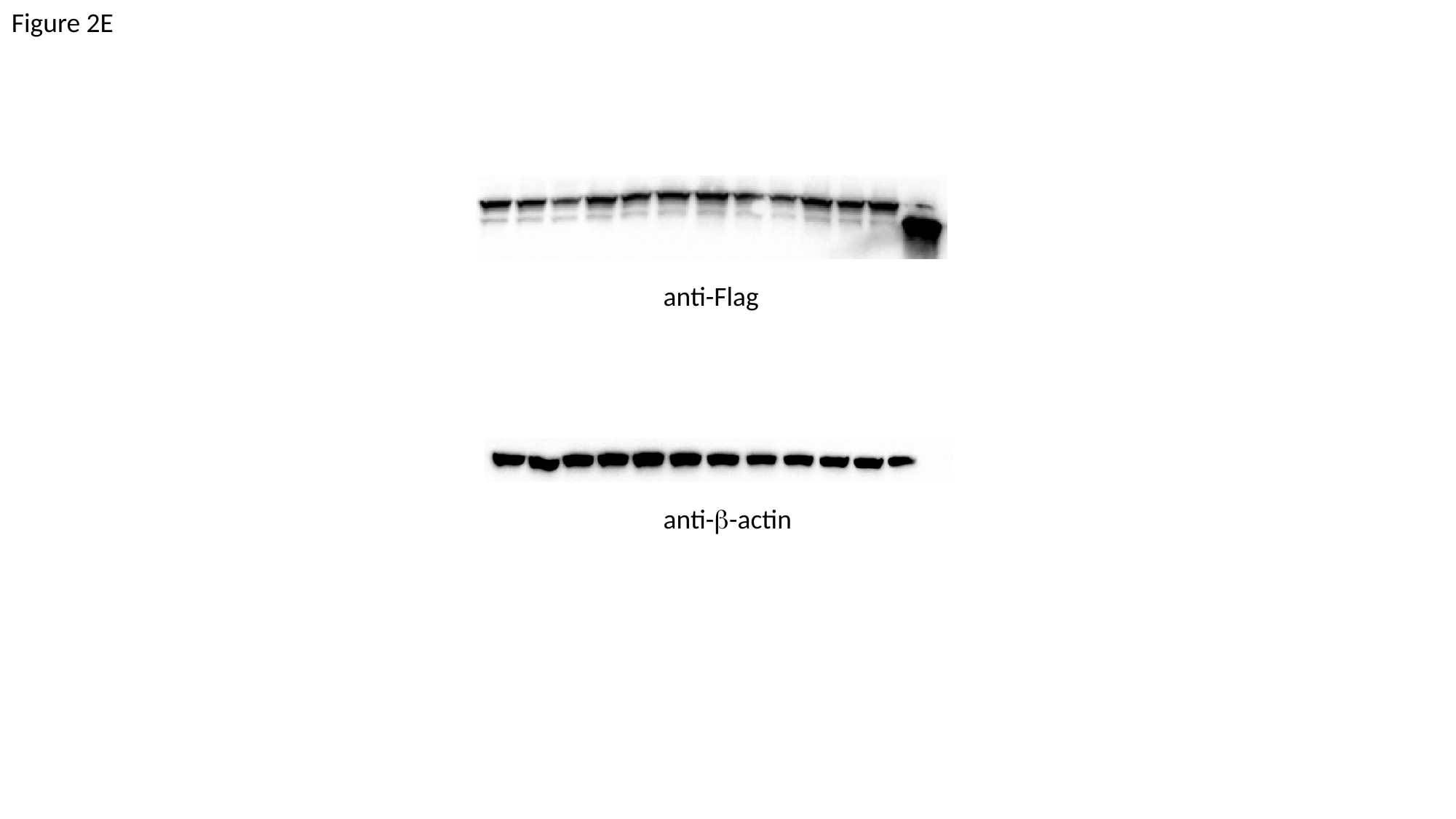

Figure 2E
anti-Flag
anti-b-actin

Supplement: Source data 2. [file elife-68445-data2.zip › unedited blot unmarked/Figure 2E unedited western blot source data unmarked.pptx]

## Slide 1
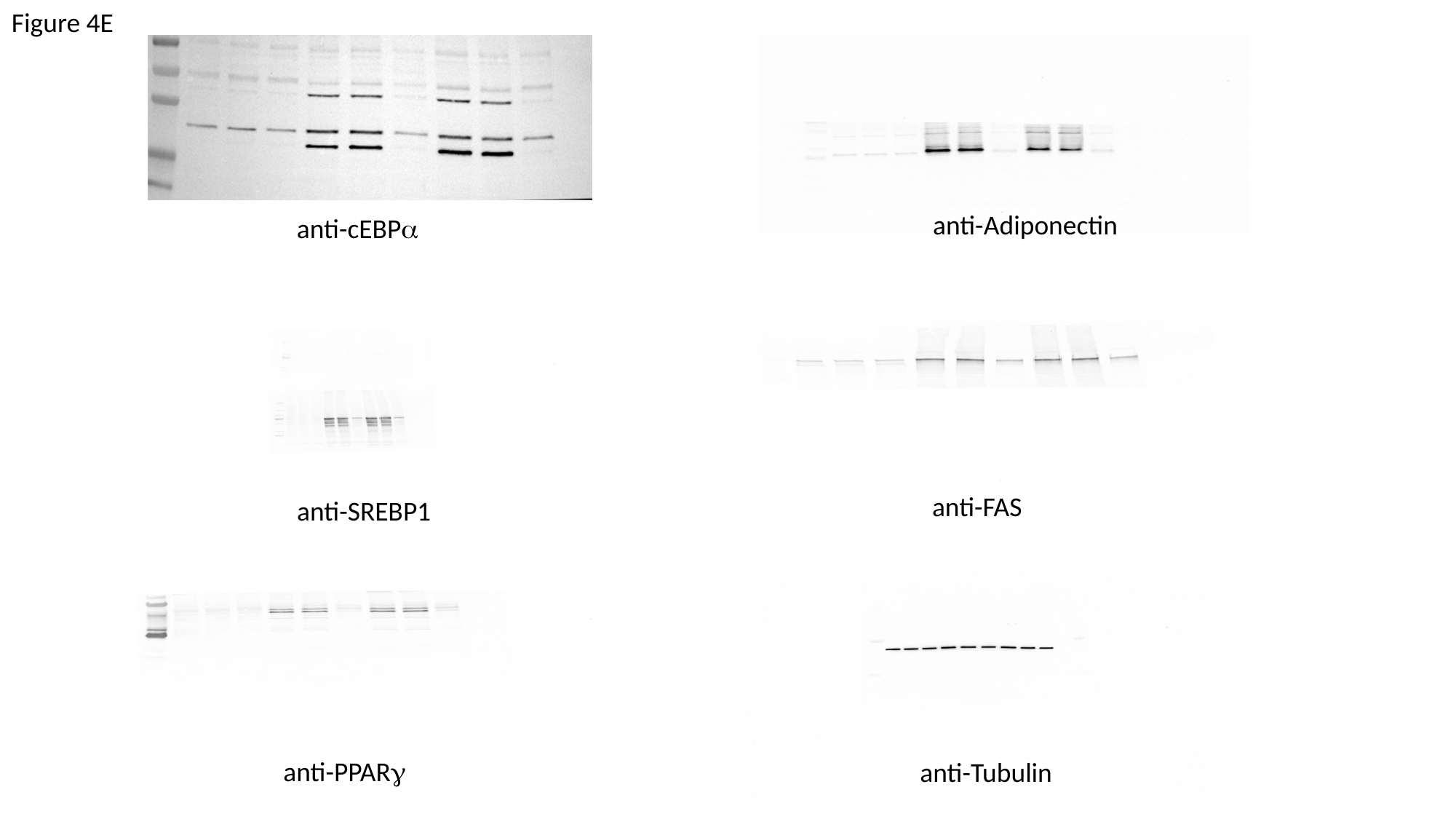

Figure 4E
anti-Adiponectin
anti-cEBPa
anti-FAS
anti-SREBP1
anti-PPARg
anti-Tubulin

Supplement: Source data 2. [file elife-68445-data2.zip › unedited blot unmarked/Figure 4E unedited western blot source data unmarked.pptx]

## Slide 1
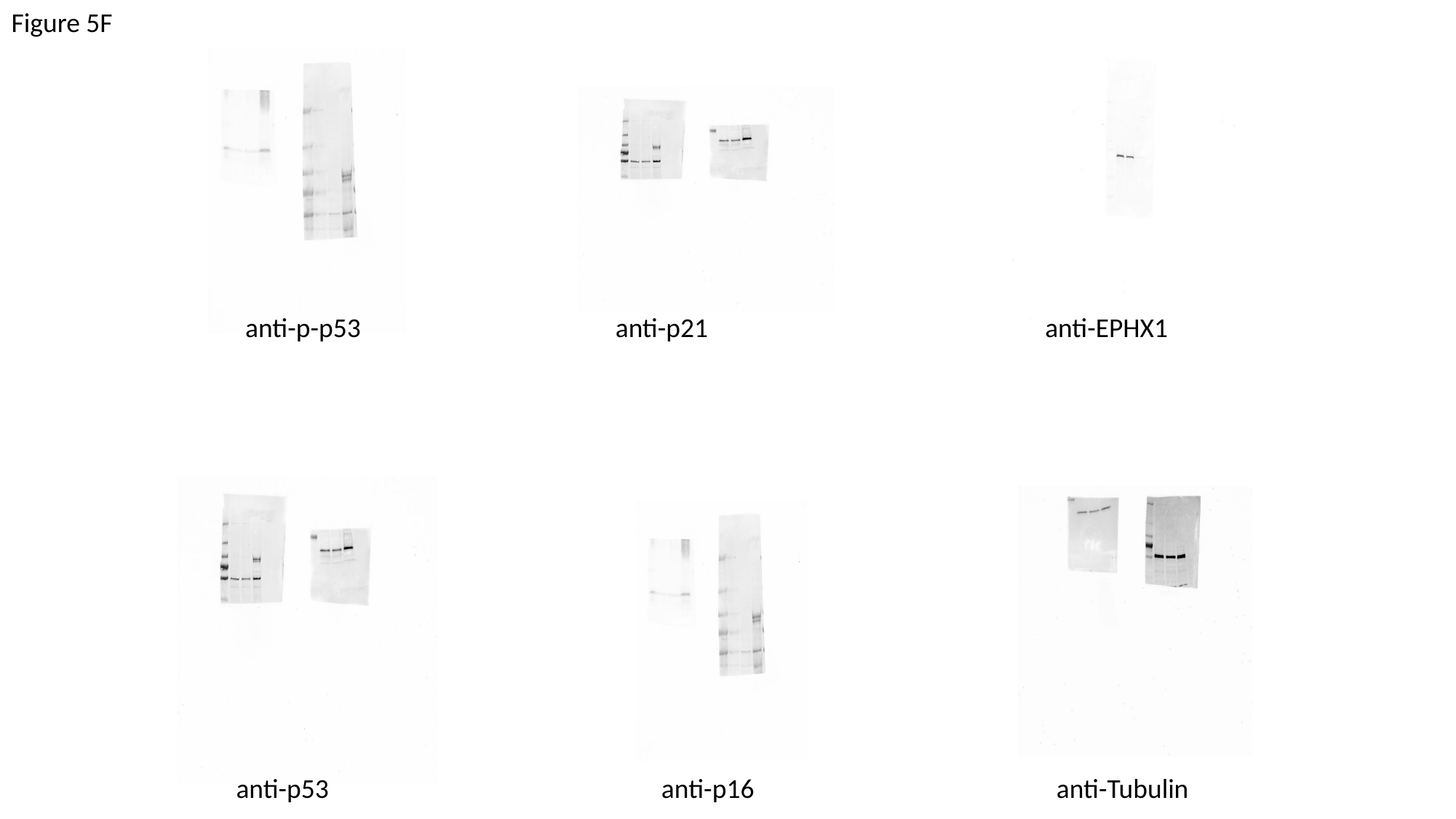

Figure 5F
anti-p-p53
anti-p21
anti-EPHX1
anti-p53
anti-p16
anti-Tubulin

Supplement: Source data 2. [file elife-68445-data2.zip › unedited blot unmarked/Figure 5F unedited western blot source data unmarked.pptx]

## Slide 1
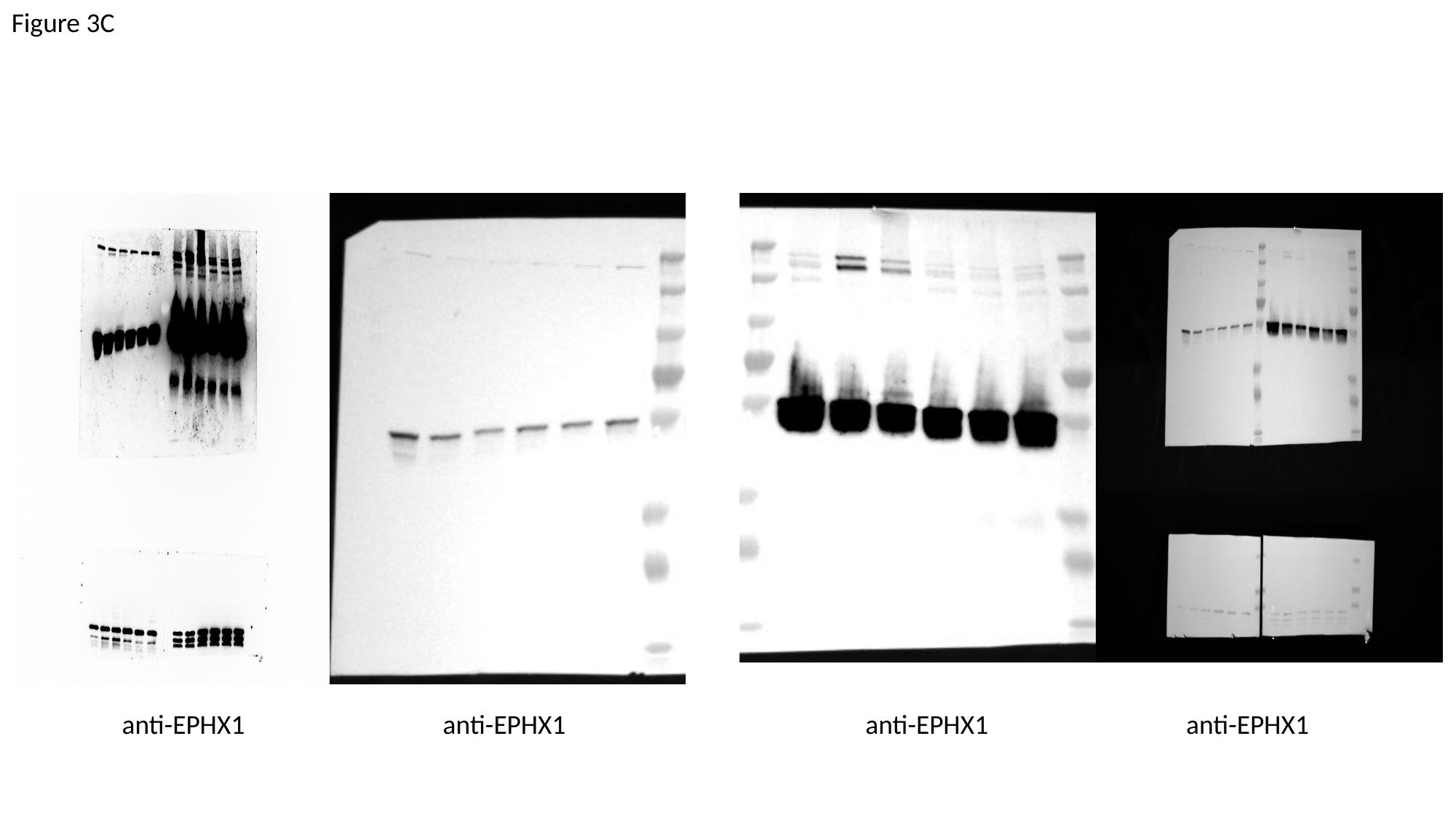

Figure 3C
anti-EPHX1
anti-EPHX1
anti-EPHX1
anti-EPHX1

Supplement: Source data 2. [file elife-68445-data2.zip › unedited blot unmarked/Figure 3C unedited western blot source data unmarked.pptx]

## Slide 1
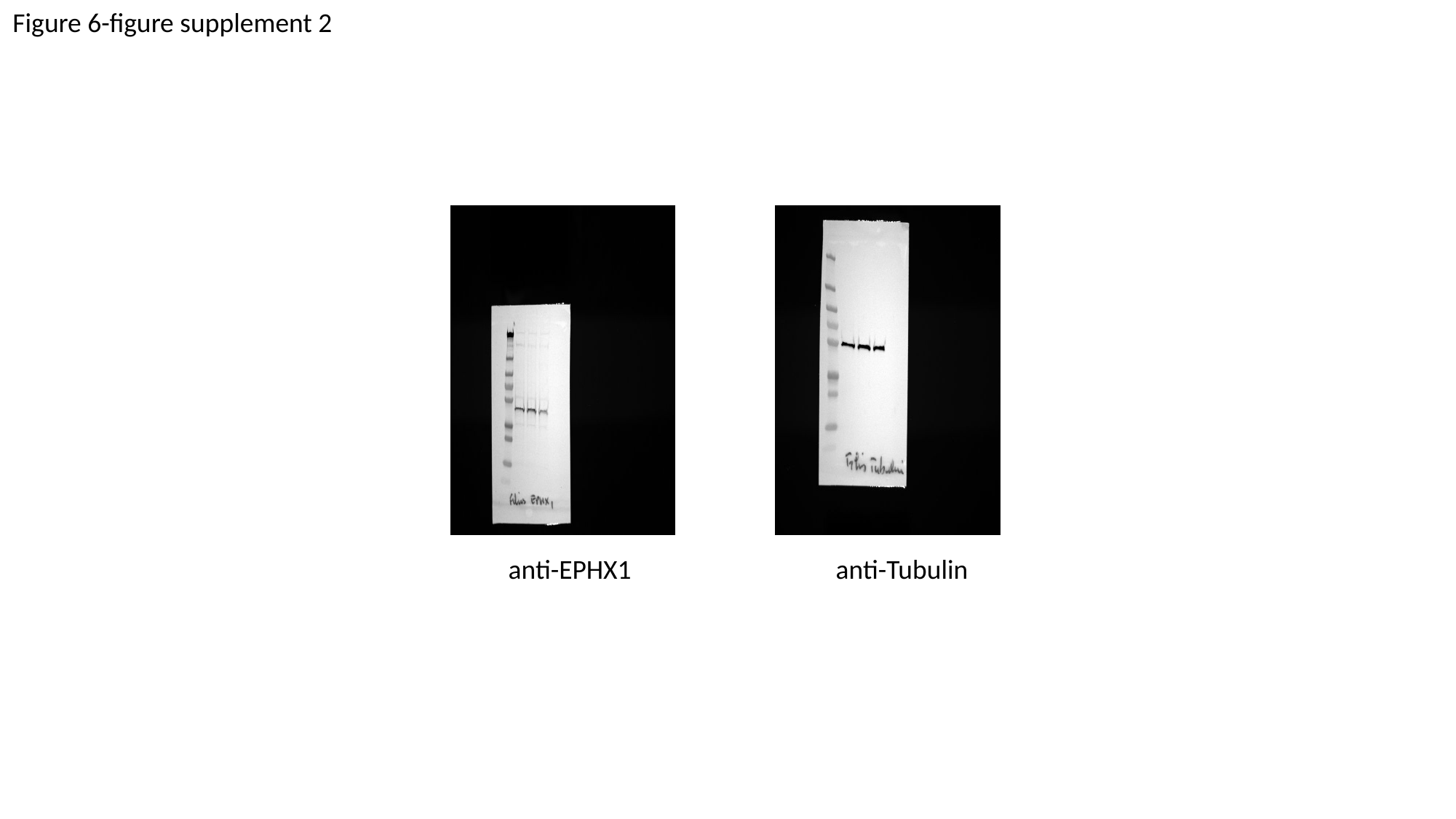

Figure 6-figure supplement 2
anti-EPHX1
anti-Tubulin

Supplement: Source data 2. [file elife-68445-data2.zip › unedited blot unmarked/Figure 6-fig suppl 2 unedited western blot source data unmarked.pptx]

## Slide 1
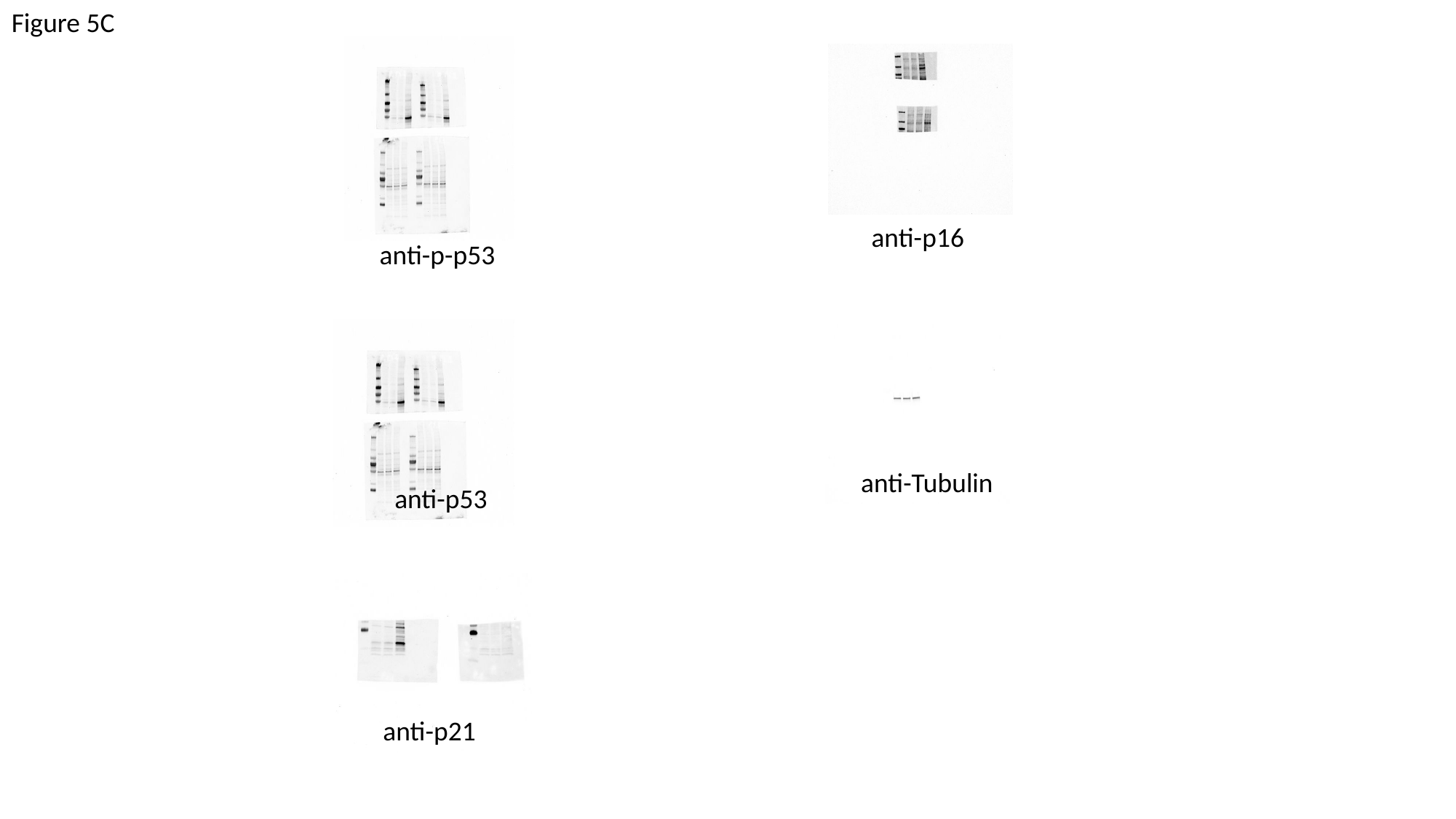

Figure 5C
anti-p16
anti-p-p53
anti-Tubulin
anti-p53
anti-p21

Supplement: Source data 2. [file elife-68445-data2.zip › unedited blot unmarked/Figure 5C unedited western blot source data unmarked.pptx]

## Slide 1
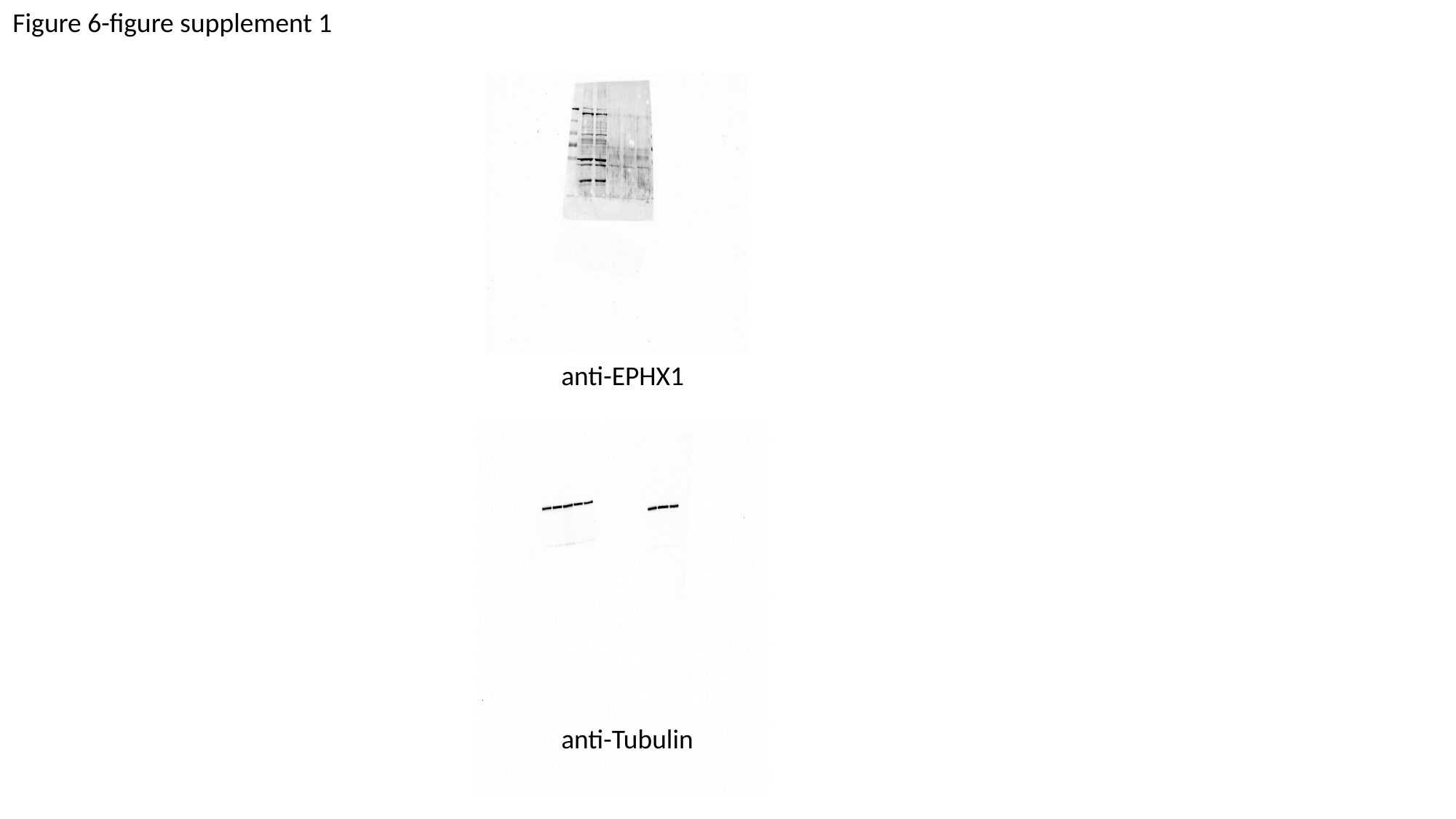

Figure 6-figure supplement 1
anti-EPHX1
anti-Tubulin

Supplement: Source data 2. [file elife-68445-data2.zip › unedited blot unmarked/Figure 6-fig suppl 1 unedited western blot source data unmarked.pptx]
